# Resilience in Resistance: The Role of Cell Wall Integrity in Multidrug-Resistant Candida

**DOI:** 10.3390/jof11040271

**Published:** 2025-04-01

**Authors:** Iván A. Banda-Flores, David Torres-Tirado, Héctor M. Mora-Montes, Gabriela Pérez-Flores, Luis A. Pérez-García

**Affiliations:** 1 Facultad de Estudios Profesionales Zona Huasteca, Universidad Autónoma de San Luis Potosí, Romualdo del Campo 501, Fracc. Rafael Curiel, Ciudad Valles 79060, San Luis Potosi, Mexico; a296711@alumnos.uaslp.mx (I.A.B.-F.); david.tirado@uaslp.mx (D.T.-T.); gabriela.perez@uaslp.mx (G.P.-F.); 2 Departamento de Biología, División de Ciencias Naturales y Exactas, Campus Guanajuato, Universidad de Guanajuato, Noria Alta s/n, Col. Noria Alta, Guanajuato 36050, Guanajuato, Mexico; hmora@ugto.mx

**Keywords:** *Candida*, cell wall, therapeutic targets, *Candida albicans*, *Nakaseomyces glabratus*, *Candida auris*, antifungal resistance, cell wall integrity pathway

## Abstract

The *Candida* species cell wall plays a pivotal role as a structural and functional barrier against external aggressors and as an intermediary in host–pathogen interactions. *Candida* species exhibit unique adaptations in their cell wall composition, with varying proportions of chitin, mannans, and β-glucans influenced by the environmental conditions and the morphological states. These components not only maintain cellular viability under osmotic, thermal, and chemical stress, but also serve as the key targets for novel antifungal strategies. MAPK signaling pathways, like the cell wall integrity pathway and the high-osmolarity glycerol pathway, play a crucial role in responding to cell wall stressors. Due to the rise of antifungal resistance and its clinical challenges, there is a need to identify new antifungal targets. This review discusses the recent advances in understanding the mechanisms underlying cell wall integrity, their impact on antifungal resistance and virulence, and their potential as therapeutic targets of *C. albicans*, *N. glabratus*, and *C. auris*.

## 1. Introduction

Candidiasis refers to a variety of infections caused by different *Candida* species, including the mucosal, cutaneous, and invasive forms. Invasive candidiasis is mainly linked to *Candida albicans* [1], followed by *Nakaseomyces glabratus* (known before as *Candida glabrata*), *C. krusei*, *C. tropicalis*, and *C. parapsilosis*, accounting for over 90% of cases [2]. The risk factors for invasive *Candida* infections include the use of broad-spectrum antibiotics, blood transfusion, central venous catheters, mechanical ventilation, parenteral nutrition, and HIV infection [3]. Azoles are the most commonly used antifungal agents for managing candidiasis due to their low-level toxicity and broad spectrum of action [4]. Additionally, caspofungin (CAS), an echinocandin derivative, is recommended as the first-line treatment for candidiasis [5]. However, in Intensive Care Units, *Candida* infections can be challenging due to the emergence of antifungal-resistant strains [6]. *Candida* species develop antifungal resistance through various mechanisms, such as mutations or a high number of copies in the genes involved in ergosterol (*ERG11*) and β-glucan synthesis (*FKS1* and *FKS2*) [7]. The overexpression of efflux pump genes (*CDR1*, *CDR2*, *SNQ2*, and *PDR6*) and transcription factors (Mrr1p and Tac1p) also contributes to resistance [8]. Additionally, the overexpression of *MKC1*, an ortholog of *S. cerevisiae SLT2*, encoding a serine/threonine mitogen-activated protein kinase (MAPK), has been identified in azole-resistant *C. tropicalis* isolates [9], playing a role in the cell wall integrity (CWI) pathway [10].

Most of the fungal cell walls are bilayered structures, with an inner layer composed of glucan and chitin and an outer layer rich in glycoproteins, with embedded proteins in both the layers [11]. This structure is essential for fungal survival, making it a target for antifungal therapy against *Candida* spp. [12]. Cell wall disruption triggers signaling cascades, particularly the CWI pathway. Some studies have shown that disrupting the CWI pathway genes and other CWI-related genes increases the susceptibility to antifungals and cell wall-perturbing agents, such as CAS, zymolyase, and calcofluor white. However, the effects may vary among *Candida* species and yeast genera. Thus, identifying new therapeutic targets in signaling cascades is a promising approach, targeting the downstream effectors, gene regulation, or translation regulation at different levels. This review discusses recent research on CWI-related pathways, the methods used in these studies, and their potential clinical implications for the development of new therapeutic strategies, specifically focusing on *Candida albicans*, *Nakaseomyces glabratus*, and *Candida auris*.

## 2. The Yeast Cell Wall: Function, Composition, Structure, and Biosynthesis

The yeast cell wall is located outside the plasma membrane and plays a crucial role in the cell’s interactions with the environment [13]. It confers protection against physical and osmotic stress, maintains cell membrane integrity throughout the cell cycle, and regulates the intracellular turgor and cell shapes [14]. The outer layer of the cell wall is composed of *N-* and *O-*linked mannoproteins connected to the inner layer by glycosylphosphatidylinositol (GPI) anchors and β-1,6-glucan linkages [15,16,17]. The inner layer of the cell wall contains β-glucan and chitin. Approximately 83% of glucan is β-1,3-glucan, while the remaining portion consists of β-1,6-glucans that play a key role in cross-linking β-1,3-glucans [18] and binding cell wall proteins (CWPs) to the β-1,3-glucan–chitin skeleton via GPI anchors [19], or through an alkali-sensitive linkage (non-GPI CWP or Pir-CWP) [20]. Chitin is a linear polymer of β-1,4-*N*-acetylglucosamine (GlcNAc) and accounts for 1–2% of the cell wall dry weight in yeast but can reach 7–15% in filamentous fungi [21]. Most of the *Candida* cell wall models consider a layered arrangement with chitin closest to the cell membrane, followed by a layer of β-glucan and mannosylated proteins attached to β-1,3-glucan via β-1,6-glucan linkages forming an outer layer [11]. Another proposed model suggests that β-glucan and chitin form intrachain hydrogen bonds and assemble into microfibrils that form a basket-like scaffold [11]. Interestingly, the *C. albicans* chitin content varies depending on its morphological phase, with hyphae containing up to three times more chitin than the yeast form [22]. However, the profile of the components in the cell wall may also differ among *Candida* species, such as the deposition of chitosan in *C. dubliniensis* chlamydospores due to a conserved pathway [19]. In addition, it has been proposed that the β-glucan chains in *C. albicans* yeasts extend into the mannan layer [11], but also glucan exists in macromolecular complexes with glycogen in *C. albicans* and other *Candida species* [23]. Furthermore, *C. albicans* can modulate the exposure of β-1,3-glucan under nutritional stress and during an immune response [24]. Interestingly, the thickness of the yeast cell wall can vary under different growth conditions. In *C. albicans* grown in a YEPD medium, it was observed that the thickness of the inner cell wall is not solely determined by β-1,3-glucan and chitin, but also by the CWPs. The way CWPs are linked to β-1,3-glucan and chitin, as well as the remodeling of these linkages and space-filling relationships, may play a role in determining inner wall thickness [11]. In contrast, the analysis of the composition and thickness of *C. auris* and *C. albicans* cell walls revealed differences in the inner and outer cell wall components that can lead to structural variations. *C. albicans* exhibited more deposition of β-glucan compared to *C. auris*, while *C. auris* showed a higher content of mannans than *C. albicans*. As a result, *C. auris* has a thicker outer cell wall, but a thinner inner cell wall compared to those of *C. albicans*, possibly due to the differences in β-glucan deposition [25]. Nuclear magnetic resonance (NMR) revealed that the mobile fraction of the cell wall, which includes mannans and β-1,6-glucan, is slightly different when comparing *C. auris* and *C. albicans* since the former showed more signals of mobile molecules [26]. Moreover, in *C. auris,* glucan has been found in a complex with glycogen, unlike *C. albicans* hyphae [23]. Additionally, *C. auris* exhibit higher levels of chitin compared to other *Candida* species [27]. Regarding *N. glabratus*, it has been observed that the mannan structures may vary strongly among strains on the length and structure of the side chains for both *N-* and *O*-linked mannans [28]. Intriguingly, glucan is not found in complexes with glycogen, unlike *C. albicans* and *C. auris* [23]. It is worth noting that the β-1,3-glucan amount may vary depending on the azole resistance phenotype. Azole-resistant strains exhibit lower levels of this molecule [29], which is associated with the upregulation of glucanases. *C. albicans* express these proteins when interacting with phagocytic cells, and this correlates with the enhanced fungal colonization of the gastrointestinal tract [24]. In addition, the analysis of cell wall components across *C. albicans*, *C. tropicalis*, and *C. guilliermondii* has shown similar levels of chitin and glucan for these species [27]. In contrast, *N. glabratus* has shown a lesser content of chitin compared with those of *C. albicans* and *C. tropicalis* [30].

**Figure 1:**
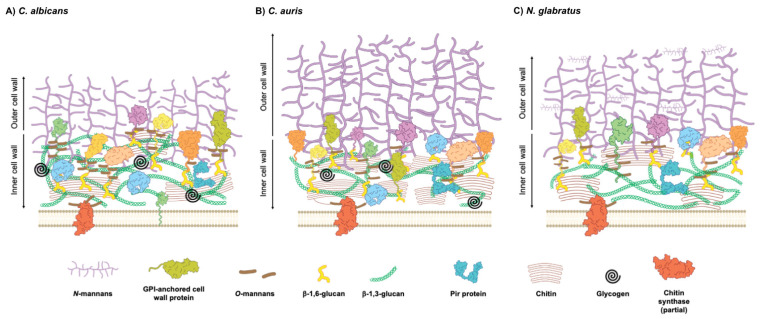
Architecture of *Candida* species cell wall. Schematic representation of *Candida* yeast cell wall, highlighting its layered structure and compositional variability among species. Outer layer consists of mannoproteins linked via β-1,6-glucan, while inner layer contains β-1,3-glucan and chitin. Proportion of these components varies depending on morphological states, environmental conditions, and species-specific adaptations. *C. albicans* (**A**) exhibits more β-glucan deposition, whereas *C. auris* (**B**) has a thicker outer mannan layer, but reduced inner wall glucan. Both contain glucan-glycogen complexes. *N. glabratus* (**C**) shows structural variations in *N-* and *O-*linked mannans and reduced chitin levels. Differences in β-1,3-glucan content are linked to antifungal resistance and immune evasion strategies. Proteins that are not specified with color code in figure represent secreted aspartic proteases, glucanases, and other related proteins. Moonlighting proteins are excluded from figure because of their transient presence in cell wall.

The cell wall proteins are a diverse group with structural and functional roles. GPI-anchored proteins are a major class of cell wall proteins. They are linked to the plasma membrane via a GPI anchor added to the protein in the endoplasmic reticulum. Also, they are covalently linked to β-1,6-glucan [31]. The analysis of 45 *C. albicans* GPI-anchored proteins revealed the wide functional role of these proteins in cell wall integrity, antifungal susceptibility, biosynthesis, and the assembly or remodeling of cell wall components [31]. Also, the recombinant GPI-anchored proteins Sap9 and Sap10, which possess aspartic protease activity, were used to cleave the GPI-CWPs of *C. albicans*, identifying Cht2 (chitinase activity during filamentous growth), Ecm33 (cell wall integrity), Pga4 (transglucosidase), and Rhd3 (unknown function; contributes to virulence) [20]. *C. auris* possesses several homologs of GPI-CWP families with similar functions as those mentioned above. It is interesting that Flo5 showed minimal sequence similarity with the *S. cerevisiae* flocculins Flo1, 5, 9, and 10, but structural similarities were found between those proteins. The homologs of other GPI-CWPs with adhesin function (Als-like adhesins, Iff/Hyr adhesins, and Flo11/Rbt1 adhesins) were found in *C. auris*, and also in *C. albicans*, *C. parapsilosis,* and *C. tropicalis* [32]. Contrasting with other *Candida* species, the *N. glabratus* genome encodes around 67 GPI-anchored proteins [33]. The *SRP1/TIP1* gene family was upregulated in vivo, and those aspartyl proteases process Epa1, a GPI-anchored adhesin, favoring fungal burden. The deletion of all six *SRP1/TIP1* gene family decreased the amount of Epa1 in the cell wall, attenuating virulence and adherence to the epithelial cells [34]. Regarding Pir proteins (proteins with internal repeats), which can bind to β-1,3-glucan directly, unlike GPI-CWPs, only two genes have been annotated in *C. albicans*, *PIR1* and *PIR32* [35]. *PIR1* has two alleles encoding Pir1. The mutated versions of the *Saccharomyces cerevisiae* ortholog Pir4 have shown that Pir-specific internal repeats are important for its covalent attachment to wall carbohydrates [36]. Intriguingly, six distinct PCR patterns of the internal repeat have been identified in *C. albicans PIR1* alleles [35]. However, bioinformatic approaches revealed nine additional proteins sharing a conserved Pir motif in this yeast [35]. In *C. auris*, two canonical Pir preproteins (Pir1 and Pir10) were found, along with Pir32. Nonetheless, since the Pir32 paralogue does not contain Pir repeats, it is more likely to be a secreted protein [32]. Exposure to echinocandins leads to changes in the cell wall architecture, and the CWP profile is not exempt to changes. In *N. glabratus*, it was observed that Pir5 displayed decreased abundance in response to CAS exposure as part of a compensatory mechanism activated by the CWI pathway, leading to an increase in chitin or mannan production [37]. The other proteins involved in the cell wall architecture and function are the moonlighting proteins. A moonlighting protein is a single polypeptide with an evolutionary conserved function, but performs extra and unrelated functions via unexpected interactions with non-canonical targets. These functions can even be different, depending on the substrate concentration, the oligomerization state, or the complex with other proteins, often occurring without structural changes in the moonlighting protein [38]. Intriguingly, moonlighting functions cannot be predicted by homology, since they do not depend on conserved motifs or domains [39]. Tsa1 (thiol-specific antioxidant-like protein) is a moonlighting protein with functions in the hyphal surface of *C. albicans*; however, it is not found in yeasts. Although this protein does not contribute to heat shock resistance or virulence, it is implicated in response to oxidative stress and is located at the cytoplasm and nucleus in both hyphal and yeast cells [40]. Recently, the *C. auris* cell wall proteome revealed an increased amount of Tdh3 (glyceraldehyde-3-phosphate dehydrogenase) in biofilms when compared to that of planktonic cells. Tdh3 is a moonlighting protein involved in glycolysis, oxidoreductase activity, and host–pathogen interaction. However, its role is yet to be discovered in this emerging pathogen [41]. Tdh3 is also found, along with pyruvate decarboxylase (Pdc11) and enolase (Eno1), in *N. glabratus*, *C. parapsilosis* and *C. tropicalis* when cultured in media simulating conditions of infection, suggesting an associated role in the pathology of these fungi [42]. Together, these studies indicate that further research is necessary to understand the specific roles of each cell wall component in the architecture of the *Candida* cell wall. A proposed model of the cell wall architecture for *C. albicans*, *C. auris*, and *N. glabratus* is depicted in Figure 1.

The biosynthesis of cell wall components occurs in various cell compartments. β-1,3-glucan is synthesized at the plasma membrane by glucan synthase. This protein complex uses UDP-glucose as a substrate within the cell and releases β-1,3-glucan to the cell wall space. Once in the cell wall, it is remodeled by hydrolases and glycosyltransferases [43].

Chitin is synthesized by a group of enzymes known as chitin synthases. Fungal chitin synthases are classified into two families, with three classes in the first family (chitin synthase I, II and, III) and four classes in the second family (chitin synthase IV, V, VI and, VII). While the main product of chitin synthase is a homopolymer with a single linkage, enzyme class VII can synthesize chitin fibrils with different architectures, possibly due to variations in intrachain hydrogen bonding or folding [20]. In *C. albicans*, four genes encode chitin synthases [19]. Among them, *CHS1* encodes a chitin synthase from class II, which is essential for cell viability [13]. Recently, it was demonstrated that the loss of Pho84, a transporter of inorganic phosphate (Pi) in *C. albicans*, is related to the decrease in detectable chitin in the cell wall. The restriction of Pi, produced by the absence of Pho84, disrupts the synthesis of UDP-*N*-acetylglucosamine, the substrate of chitin synthases [44]. In *C. auris*, *PHO84* is upregulated when cells are cultured with human peripheral blood, thus being implicated on the survival in the host [45]. This protein is also relevant for *N. glabratus*, as up to 10% of genes regulated by Pho84 are implicated in cell wall organization [46].

Mannans are synthesized in the endoplasmic reticulum (ER) and Golgi complex by mannosyltransferases, which glycosylate oligomannosyl residues using either Dol-P-Man or GDP-mannose as substrate [19]. The other enzymes involved in mannoproteins synthesis include glycosylhydrolases and transglycosidases. The majority of mannoproteins in the cell wall are GPI-modified proteins, accounting for approximately 88% of the total [27]. The GPI anchor is preassembled in the ER, where GlcNAc-phosphatidylinositol undergoes deacetylation, triple mannosylation, and decoration with 2–3 phosphoethanolamine groups at the third mannose. The phosphoethanolamine then anchors to a protein with a GPI anchor signal sequence in the ER lumen, resulting in the formation of a GPI-anchored protein [47]. In *C. albicans*, two homologous mannosidases Dfg5 and Dcw1 were related to the proper activity of the CWI pathway and pathogenesis [48]. In *N. glabratus*, this protein may be related to similar functions through activation via the calcineurin signaling pathway [49]. In addition to this, this mannosidase seems upregulated upon exposure to CAS in *C. auris*. [50]. The effect of this glycoprotein on cell wall biogenesis is related to the regulation of MAPKs in *Saccharomyces cerevisiae* and *C. albicans*. In the latter, the lack of Dfg5/Dcw1 correlates with low levels of gene expression related to the CWI pathway [48].

## 3. Pathways to Preserve Cell Wall Integrity

Due to its location and its vital role in interacting with the environment and hosts, as well as mediating biological functions, such as morphology, permeability, and protection [51], the fungal cell wall is responsible for sensing and responding to different types of stressor. To adapt to and survive external stimuli, yeasts utilize MAPKs as a key mechanism. The MAPK signaling pathways encompass a cascade of three kinases (MAPK kinase kinases [MAPKKK], MAPK kinases [MAPKK], and MAPK) that activate transcription factors [52]. The CWI pathway is the primary MAPK-mediated pathway responsible for cell wall reconstruction, widely described in the literature on *S. cerevisiae* and *C. albicans*. Additionally, the other MAPK pathways such as the high-osmolarity glycerol (HOG) pathway, Extracellular signal-Regulated Kinases 1/2 (ERK1/2) signaling cascade, and the Cek1-mediated pathway also contribute to fine-tuning cell wall restoration [53].

The CWI pathway possesses membrane-spanning mechanosensors, such as Wsc1, Wsc2, Wsc3, Mid2, and Mtl1, with a nanospring-like extracellular region that extends into the cell wall [52]. These receptors detect disturbances in the cell wall, triggering downstream MAPK modules (Bck1, Mkk1/Mkk2, and Slt2) to initiate transcription machinery for cell wall remodeling. Interestingly, the deletion of the *BCK1*, *MKK1*, and *SLT2* genes in *S. cerevisiae* results in viable cells, even in the absence of cell wall stress, suggesting that the upstream MAPKs (Pkc1) may activate other targets outside the CWI pathway [52]. Several research groups have conducted screenings and gene engineering to uncover the interactions among the pathways involved in CWI and to identify new genes regulated by these pathways. Some screening studies have identified cell wall-related mutants sensitive to the K1 and K2 toxins, Congo red (CR), CAS, zymolyase, the freeze–thaw cycle, and heat stress [54]. In addition, *C. albicans RAP1* deletion, which is involved in cell replication, has been associated with changes in cell wall properties. The *rap1*Δ/Δ mutant strain exhibited susceptibility to CR and calcofluor white (CFW), but less susceptibility to tunicamycin, possibly due to increased cell wall mannan, chitin, and glucan contents, along with the upregulation of *CHS8*, *XOG1*, and *PHR1* expression. Furthermore, it was demonstrated that *RAP1* deletion led to increased Mkc1 phosphorylation when compared to that of the wild-type and the reintegrated strains [55].

New insights into the role of genes involved in the aromatic amino acid biosynthesis have demonstrated that the upregulation of *C. albicans ARO1* impacts CWI. The doxycycline (DOX)-mediated silencing of *ARO1* revealed increased susceptibility to CFW, CR, and zymolyase, correlating with a reduced carbohydrate content. Interestingly, *ARO1*-silenced strains showed CAS resistance, which was reversed upon DOX addition. Furthermore, a decrease in the Mkc1 phosphorylation level was observed when *ARO1* was silenced, suggesting a potential role in CAS resistance. Aro1 or its products may negatively regulate β-glucan synthase. Alternatively, the decrease in the level of synthesis of aromatic amino acids may induce β-glucan synthase to increase resistance and survival against metabolic deficiencies. Further research is needed to elucidate the exact mechanism by which Aro1 influences CAS resistance and its potential role in the CWI pathway [56].

In addition, *SSK1* (a two-component response regulator) and *HOG1* (MAPK) disruptions in *C. auris* alter its membrane architecture and susceptibility to various cell-wall-perturbing agents, such as amphotericin B (AMB), CAS, caffeine, and sodium dodecyl sulfate (SDS). The relationship between the presence of Ssk1 and Hog1 activation was demonstrated, as *SSK1* deletion led to the significant downregulation of Hog1 phosphorylation. This suggests that the interruption of gene expression involved in fungal biochemistry activities may reveal an expanded network of proteins that activate enzymes to preserve CWI during different fungal life stages [57].

Previous research has described the interplay between the CWI and HOG pathways. Recent studies manipulating genes in both the signaling cascades have shown that Sko1, a transcription factor from the HOG pathway, is phosphorylated not only by Hog1, but also by Rlm1, a CWI pathway transcription factor. This suggests that Sko1 regulation may be independent of HOG pathway signaling. However, the mechanism by which Rlm1 activates Sko1 remains unclear [58]. Other pathways, such as the cAMP-PKA signaling pathway in *C. auris*, also play a role in CWI maintenance. In experiments with CFW and CR, *C. auris* strains lacking *TPK2* and *CYR1* showed reduced growth, while a strain lacking *TPK1* showed increased growth. The deletion of *BCY1* increased resistance to CFW and CR. Tpk1 and Tpk2 promote the expression of genes involved in chitosan and chitin biosynthesis, but *TPK1* deletion did not affect the transcription of certain chitin synthase genes (*CHS4*, *5*, *7*, and *8*). The roles of *TPK1* and *TPK2* appear to be opposite, and *BCY1* deletion reduced the transcription levels of the same *CHS* genes. However, the reason for the enhanced resistance to CFW and CR in the *bcy1*Δ mutant strain is not yet understood. Further exploration of the connections between the cAMP-PKA pathway and other pathways may provide insights into the role of this signaling pathway in maintaining CWI [59].

**Figure 2:**
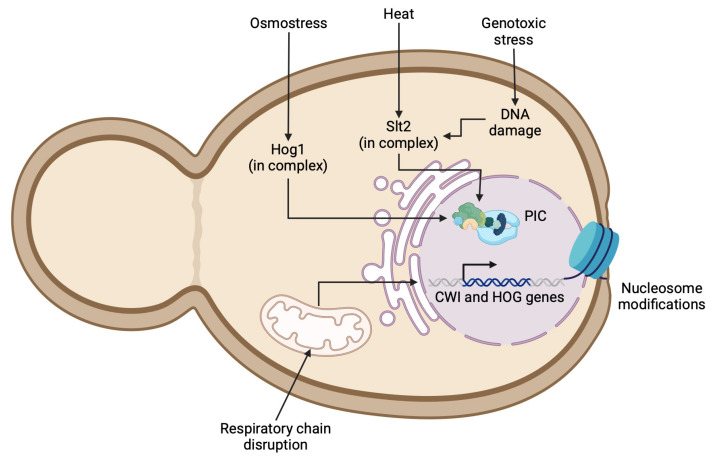
Regulation of CWI and HOG pathway gene expression under stress conditions. Schematic representation of cellular mechanisms regulating expression of cell wall integrity (CWI) and high-osmolarity glycerol (HOG) pathway genes under stress conditions. Figure illustrates how osmotic stress, heat, and genotoxic stress activate key protein complexes containing Hog1 or Slt2, leading to formation of pre-initiation complex (PIC). Additionally, mitochondrial respiratory chain disruptions and nucleosome modifications are depicted as contributing factors in gene regulation.

The MAPKKs are dual-specific protein kinases that phosphorylate and activate MAPKs. The MAPKKs Mkk1 and Mkk2 phosphorylate the T190 and Y192 residues within the T-E-Y motif of the Slt2 activation loop. Another mechanism of Slt2 activation may be driven by genotoxic stress, which induces DNA damage and activates Slt2 through the proteasomal degradation of Msg5, a phosphatase specific for Slt2 [60]. Once the CWI or HOG pathway phosphorylates their last upstream MAPK, Slt2 and Hog1, respectively, these kinases activate the transcription factors involved in yeast cell wall remodeling (Figure 2). In the CWI pathway, Rlm1 is the main transcription factor. Gene expression in eukaryotes involves chromatin remodeling to bind transcription factors. Sanz et al. (2022) identified the essential genes using a reporter plasmid (MLP1p-MLP1-GFP) and uncovered a broad transcriptional response mediated by the CWI pathway, including genes involved in chromatin remodeling related to Slt2 phosphorylation [55]. SBF (Swi4/Swi6), another CWI transcription factor, regulates the expression of a small group of genes, such as *FKS2*, *CHA1*, YLR042C, and *YKR013W*, and requires the interaction between activated Slt2 and Mlp1 in association with the Pol*II* and Paf1C to prevent premature termination by the Sen1-Nrd1-Nab3 complex [61]. This observation suggests that Slt2, as well as Hog1, is important both for the initiation and the elongation during the transcription of cell wall genes (Figure 2) [62].

In *C. albicans*, the null mutants of the non-essential component Rsc4 and the novel subunit Nri1 from the Remodels the Structure of Chromatin complex (RSC) exhibit sensitivity to cell-wall-perturbing agents and osmotic stress [63]. It has been reported that the chromatin remodeling complexes SWI/SNF and SAGA may bind to Rlm1 to regulate the promoters of the CWI pathway genes. Nonetheless, the exact role of Rlm1 and other transcription factors in the activation of all screened CWI pathway-activated genes is not fully understood [64]. Similarly, in the HOG pathway, recent studies have shown that Spt20, a protein from the SAGA complex in *C. albicans*, plays a structural and functional role. It was observed that Spt20 does not induce Hog-dependent genes, such as *HGT10*, *SKO1*, *CAT1*, and *SLP3,* in the presence of 1M NaCl in *spt20*Δ/Δ mutants. Additionally, reduced levels of phosphorylated Hog1 were observed in this mutant, indicating the importance of Spt20 in the yeast response to osmotic stress [65]. Moreover, nucleosome composition appears to be a key regulator of cell wall genes (Figure 2). It has been suggested that the histones H3, H2, and H1 positively regulate *RHO1,* but downregulate *ROM2*, genes enconding a GTPase and a guanine nucleotide exchange factor, respectively [66]. Strains lacking *HTZ1*, a gene encoding a protein involved in the histone exchange of H2A.Z for histone H2A, demonstrate sensitivity to cell wall stress by CFW [67]. Genetic analysis revealed that the histone residues H4-S40 and T30 are phosphorylated in response to heat and osmotic stress in *S. cerevisiae* [68]. In *C. auris*, the disruption of acetyltransferase Gcn5 of the SAGA complex revealed its role in driven pathogenesis in mice and developing antifungal resistance, positively mediating the efflux pump genes *CDR1*, *SNQ1* and *MDR1* and genes related to cell wall homeostasis and resistance to CAS (*FKS1*, *FKS2* and *CAS5*, respectively) [69]. Intriguingly, the chromosomal rearrangements in *C. auris* display clade-specific karyotypes, correlating with the different repertoires of surface proteins like adhesins and other GPI-anchor proteins and modifications in the cell wall stress response of emerging *Candida* species. In addition, *N. glabratus* lacking Snf2 from the SWI/SNF complex had a higher level of expression of IL-1β during phagocytosis and the overexpression of surface *EPA1* adhesin and diminished proliferation in macrophages. Moreover, *snf2*Δ strain was more sensitive to osmostress, a low pH, CR, fluconazole, among others [70]. In *N. glabratus*, the deletion of genes involved in histone modification, such as *ADA2*, *GCN5*, and *RPD3*, increase the susceptibility to antifungal stressors and reduce the virulence in infection models, highlighting the importance of epigenetic regulation in pathogenicity [71]. Further research is needed to elucidate the role of histone remodeling and chromosomal rearrangements. The proteins involved in such processes could potentially serve as antifungal targets, as it has been suggested for *C. albicans* and *N. glabratus* [66,71,72].

At the post-transcriptional level, several RNA-binding proteins (RBPs) can influence the activity of genes involved in cell wall synthesis. In *S. cerevisiae*, Nab6 has been found to bind to transcripts from *SCW4*, *PIR1*, and *MRH1*, among others, leading to positive modulation in budding yeasts. In contrast, Mrn1 plays the opposite role [73]. *SSD1* encodes an RNA-binding protein that regulates the transcription of various CWPs. In *Candida albicans*, the deletion of *SSD1* resulted in increased sensitivity to cell wall stressors and a slight sensitivity to heat stress, whereas *N. glabratus* lacking *SSD1* did not show increased sensitivity to either condition. Additionally, the deletion of *SSD1* did not affect the localization of Sun4, a cell wall glucanase [74]. This finding contrasts with another study on *N. glabratus*, which reported that *SSD1* deletion altered the localization or function of Fks1, the catalytic subunit of β-glucan synthase, while the Fks2 mRNA and translation levels appeared to increase [75]. Altogether, these data suggest that these proteins are regulated in a differential manner. However, the exact mechanisms of Nab6, Mrn1, and Ssd1 in the regulation of transcripts remain unknown [73,75,76]. In *S. cerevisiae*, *TPK1* is upregulated under heat stress, and mRNA is assembled into discrete granules. However, *TPK1* translation is restored at 25 °C. The Tpk1 protein may bind to the Cas5 promoter (a CWI transcription factor) to downregulate the CWI pathway. These findings suggest there is a *TPK1* post-transcriptional modification that allows for the CWI pathway to be followed, but at low temperatures, Tpk1 negatively regulates this pathway [77]. Additionally, *C. auris* lacking *TPK1* has shown increased growth under heat stress at various temperatures [60].

The negative regulation of the CWI pathway also occurs at upstream levels through post-translational modifications of proteins by phosphatases (Ptp2, Ptp3, Msg5, and Sdp1), the ubiquitination of Pkc1, and the negative feedback of Rom2 by Slt2, ultimately modulating the transcription of the CWI pathway genes [62].

## 4. Adaptive Remodeling of Yeast Cell Walls Under Antifungal and Environmental Stress

As mentioned earlier, stress on yeast cell walls can trigger highly regulated intracellular signaling systems to enhance the structural resistance of the wall and cell viability in adverse environments. Yeasts utilize various sensors embedded in the plasma membrane to detect a wide range of stressors, such as antifungals, salinity, and nitrogen variations, among others [78].

While the availability of antifungals is currently limited, in recent years, several compounds, such as echinocandins, natural products, nucleosides, and synthetic molecules, among others, have been proven to disrupt fungal cell walls, including those of *Candida* species. These compounds target enzymes like β-1,3-glucan synthase and β-1,6-glucan synthase, chitin synthase, chitinases, mannans, Gwt1, and GlcN-6-P synthase [79]. Exposure to echinocandins can trigger *Candida* species to increase chitin expression in their cell wall, leading to echinocandin tolerance. The development of resistance to various drugs, including echinocandins, polyenes, and azoles, suggests the involvement of alternative signaling networks that activate the stress response pathways. Upstream kinases like Pkc1, Bkc1, and Mkc1, which are clients of Hsp90, can be targeted pharmacologically to enhance antifungal therapy through synergistic effects. Inhibiting Hsp90 disrupts stress response signaling cascades, making *C. albicans* more susceptible to antifungals [80]. Exploring chaperone proteins in antifungal tolerance is intriguing, as targeting proteins in stress response signaling pathways may result in phenotypic differences compared to the wild type. For example, a *Cryptococcus neoformans* mutant lacking *BKC1* showed no essential role in activating the CWI response in cells exposed to CAS, with a similar minimum inhibitory concentration to that of the wild-type cells [52,81]. Conversely, the deletion of the cell wall maintenance genes *SLT2*, *YPK2*, *SLG1*, and *YPS1* in *N. glabratus* increased susceptibility to micafungin and CAS in vivo and in vitro [13]. In *C. albicans*, Hog1 does not appear to play a significant role against micafungin, as a *hog1*Δ/Δ mutant did not exhibit growth defects in the presence of this antifungal [82]. Therefore, targeting chaperone functions may be a valuable approach due to variations in the antifungal resistance phenotypes. Interestingly, poacic acid (PA), which binds to the β-1,3-glucan network, can inhibit the activity of the cell wall-remodeling enzymes Crh and Gas. However, the higher cell wall β-1,3-glucan content and the activation of the CWI pathway by PA may impact its antifungal activity [83].

Other cell-wall-perturbing conditions can activate signaling cascades. Water activity in the environment (a measure of the availability of water in a substance for microbial growth, chemical reactions, and enzymatic activity) is dynamic and can change constantly, leading to variations in the osmolyte concentration between the environment and the yeast cells. Osmoregulation is the process by which cells sense and counteract osmotic challenges, making it crucial for cell survival [84]. Changes in osmolarity can negatively impact the growth and viability of yeasts, causing cell shape rearrangements, such as shrinkage and the loss of water [85]. To successfully regulate osmostress, yeasts rely on sensing and signaling machinery whose response depends on the carbon source and concentration, resulting in physiologically altered responses. The HOG pathway in *S. cerevisiae* is glucose-dependent and essential to glycerol biosynthesis. Interestingly, glycerol accumulation in yeast cells under hyperosmotic conditions can lead to cell wall disruption [86]. The HOG pathway activation during osmostress occurs over several hours, while short-term (in seconds) volume alterations are regulated by other mechanisms involving aquaporins and yeast monovalent transport systems [87]. The yeasts must stabilize their cell membrane in response to osmotic stress. As a commensal organism, *C. albicans* must adapt to dehydrating conditions in the large intestine, similar to conditions observed in glomeruli, where *C. albicans* become pathogenic [88]. In *C. albicans*, the Hog1 MAPK plays a critical role in lipid homeostasis under NaCl osmostress conditions. A *hog1*Δ/Δ mutant in *C. albicans* accumulates intracellular lipids, leading to decreased viability [88]. In addition, the *Cryptococcus gattii HOG1* null mutant exhibits reduced growth in a medium supplemented with high concentrations of NaCl and KCl, along with a decreased capsule, melanin production, and virulence [89]. In *C. auris hog1*Δ mutants, the enrichment of mannans was observed, highlighting the role of *HOG1* in cell wall remodeling in *Candida* species (Figure 2). Interestingly, *HOG1* deletion resulted in different resistance profiles against AMB and CSF in *C. auris*. The AR384 isolate was sensitive to AMB, while the AR389 and 1184/P/15 strains showed increased basal tolerance to AMB and CAS [58]. In contrast, *C. albicans hog1*Δ/Δ mutants showed increased susceptibility to AMB [90]. Additionally, it was demonstrated that *HOG1* in *N. glabratus* is required for iron homeostasis and gut epithelial adherence, regulating colonization in a mouse gastrointestinal model [91].

Yeast adaptation to different host niches also involves proper mitochondrial function. The inhibition of single complexes of the respiratory chain in *C. albicans* correlates with cell wall changes, exposing β-glucans (Figure 2) and enhancing phagocytosis by macrophages [92]. Some natural products are being tested to develop new antifungal agents, such as naringin, a flavonoid that may induce the production of reactive oxygen species in *C. albicans*, interfering with Ca^2+^ signaling and leading to apoptosis [93]. Importantly, the loss of mitochondrial function in *N. glabratus* has been associated with increased azole resistance and enhanced virulence in a murine infection model [94]. However, in *C. auris*, mitochondrial dysfunction induced by pyrvinium pamoate (an FDA-approved antiparasitic drug) led to an increase in the level of phagocytosis in vitro [95].

Nitrogen homeostasis plays a role in processes involving cell wall remodeling. The Cek1-MAPK pathway can be activated by low-level nitrogen-derived stress, leading to mycelium formation through the phosphorylation of the Chp1 and Ace2 transcription factors [96].

Yeasts also respond to physical forces, including compressive, tensile, shear, bending, and torsion forces [97]. External forces can destabilize the growth machinery during polarized growth. Mechanical stress is sensed by the Wsc receptor family [98]. When pressure is applied to the cell wall, *S. cerevisiae* Wsc1 receptors cluster at the sites of force application, recruiting downstream effectors of the CWI pathway [97]. During fungal penetration to host tissues such as epithelial barriers, *C. albicans* hyphae resist compression forces by depolarizing Rho1 [99]. Further research is needed to understand the implications of mechanical stress during tissue invasion by fungal pathogens. Altogether, these data suggest that downstream effectors of the CWI pathway and the other pathways involved in maintaining CWI may be potential targets at different stages of infection.

## 5. Experimental Approaches and Techniques in Studying Cell Wall Integrity

Studying the changes in the cell wall caused by perturbing agents requires a wide variety of techniques to elucidate structural and chemical modifications. This knowledge can enhance our understanding of biology and host–pathogen interactions in yeasts, such as those in the *Candida* genus. This section will explore current techniques used to investigate CWI in yeasts, with a focus on the *Candida* species.

### 5.1. Microscopy and Molecular Trafficking Methods

Recent studies using cryo-electron tomography and structural modeling have shown that chitin in the *C. albicans* cell wall is not an isolated layer, but rather a scaffold between mannans, glucans, and membrane or cell wall proteins [11]. In addition, this technique has proven useful to reveal structural details of both the cell wall and ribosomes in *C. auris* [100,101]. Also, it has been used for elucidating the structure of membrane proteins in *N. glabratus* like β-1,3-glucan synthase [102,103,104]. Cryo-electron tomography allows for the 3D modeling of cellular structures, but it requires high-quality samples thinned to <1 μm and the use of fluorescence techniques to locate the region of interest. Additionally, alignment errors can lead to the discarding of half of the tomograms [105].

Neeli-Venkata et al. (2021) utilized microchambers and Fluorescence Recovery After Photobleaching (FRAP) to visualize the clustering of Wsc1 at the *C. albicans* membrane and the topological localization of the downstream effectors of the CWI pathway under mechanical stress conditions [97].

Recently, Raman spectroscopy exhibited a good performance in the diagnosis of *C. auris* based on the unique spectral fingerprints of structural biopolymers, such as glucans, chitin, and mannans. Intriguingly, *C. auris* Clade 1 showed two different fingerprints, suggesting cell wall plasticity [106].

### 5.2. Molecular Biology Approaches

The deletion of genes implicated in maintaining CWI remains a powerful method for characterizing the functional role of effectors. For instance, the deletion of *C. albicans SPT20* through double homologous recombination was used to study the effects on null mutants under osmotic stress [65]. Gene expression studies have provided insights into the targets of key transcription factors using Chromatin Immunoprecipitation, followed by a sequencing (ChIP-seq) methodology. For example, Marienela et al. (2020) utilized epitope-tagged Sko1-V5 and Rlm1-V5 to identify the spectrum of *C. albicans* genes regulated by these factors in the HOG and CWI pathways, respectively [59]. Furthermore, studying cell wall biosynthesis at the transcriptomic level involves the detection of protein–RNA interactions in vivo. The cross-linking analysis of cDNAs (CRAC) entails UV cross-linking, protein purification under denaturing conditions, partial RNA digestion, DNA library preparation, and sequencing [107]. Using CRAC, Bresson et al. (2023) identified the regulatory role of *S. cerevisiae* Mrn1 and Nab6 in cell wall synthesis [73]. These methods can help uncover the genes activated by other transcription factors in the CWI pathway and other pathways related to CWI, as well as how those genes are regulated at the post-transcriptional level. Other techniques, such as Assay of Transposase Accessible Chromatin (ATAC-seq), can provide information about transcription factor binding. Unlike ChIP-seq, ATAC-seq does not require a high input of cells. Moreover, ChIP-seq is limited to acquiring information on one transcription factor per assay and is dependent on the availability of antibodies [108].

In summary, the integration of advanced microscopy techniques with molecular biology approaches provides a comprehensive view of the yeast cell wall structure and the regulatory networks. This combined approach not only deepens our understanding of cell wall integrity and its dynamic modifications in response to stress, but also sheds light on the mechanisms underlying host–pathogen interactions in *Candida* species. Ultimately, these insights are crucial for the development of innovative antifungal strategies and for improving therapeutic outcomes against fungal infections.

## 6. Clinical Implications

The candidiasis morbidity and mortality rates have increased in recent years due to conditions such as HIV infection and cancer therapies that lead to immunocompromised states in patients [109]. Although *C. albicans* is the most clinically significant pathogen in blood stream invasions and fungal invasive infections, distribution studies highlight that non-*C. albicans* species represent a global distribution of 62.8%, with *N. glabratus* being the most common of these species, with a distribution ranging from 7.8 to 30%, accordingly to hospital-based data obtained between 2010 and 2017 [110]. However, the distribution changes are slightly different among countries [111].

Echinocandins target the β-1,3-glucan synthase and have been effective in managing *Candida* species with low rates of resistance. However, *N. glabratus* may exhibit resistance rates of 3–15% [112]. Similarly, the emerging pathogen *C. auris* is showing increasing resistance to multiple antifungals, including echinocandins [79,113]. The rise of antifungal resistance represents an important challenge that limits the availability of chemotherapies. Interestingly, the mortality rates do not differ between patients with non-*albicans* and *C. albicans* candidemia resistant to azoles, emphasizing the importance of addressing antifungal resistance [114]. Hence, there is a need to deep into molecular variations among the genes involved in antifungal tolerance (Table 1). The strains of *C. albicans* possess some mutations conferring resistance to multiple antifungal classes. Some of the genes involved in resistance acquisition are *ERG3*, *ERG11* (azole resistance), and *FKS1* (echinocandin resistance) [115]. Also, *C. auris* is a multidrug-resistant emerging pathogen with innate mutations conferring resistance to various antifungals, including azoles, polyenes, echinocandins, and fluorocytosine [113]. Resistance can arise from different factors such as drug prophylaxis, agricultural antifungal use, suboptimal drug administration, non-linear pharmacokinetics, and poor patient compliance [116]. The mutations include increased expression levels of *CDR1* (ABC transporter); *MDR1* (MFS transporter); *TAC1B*, *MRR1A* (efflux pump regulation); and *FKS1, ERG1*, *ERG2*, *ERG6*, and, *ERG13* [117]. Interestingly, the conformational variability of *C. auris FKS1* and *ERG11* correlates with drug resistance [118]. Despite resistance to major antifungal classes in clinical settings [115], CAS remains the primary drug for treating candidiasis, particularly when caused by *C. auris*. Intriguingly, resistance to echinocandins and polyenes may reduce virulence in *Candida* species, but it increases with azole resistance [115]. However, the hosts may experience niche-specific events where mutations in resistance genes attenuate virulence in one niche, but enhance it in others [115]. Similar to *C. albicans* and *C. auris*, *N. glabratus* possess mutations or highly express the resistance genes mentioned above, conferring tolerance to azoles, polyenes, and echinocandins like CAS and micafungin [119]. Intriguingly, colonies isolated from blood cultures showed genotypic diversity, revealing that some populations are resistant to antifungals, which may lead to failure in the management of *N. glabratus* candidemia [120].

Importantly, the geographic distribution of *Candida* species also correlates with different susceptibility profiles; for example, more resistance to azoles compared with that of echinocandins has been observed in Ibero-America, Europe, and Asian Pacific regions [8]. Regarding *C. auris*, Clade I (South Asia) and Clade III (South Africa) have a distinctive resistance pattern, indicating that both the clades are resistant to azoles, although the point mutations in *ERG11* conferring resistance to fluconazole are clade-specific: K143R and Y132F in Clade I and F126L in Clade III [121]. Intriguingly, resistance patterns can be different among clades and geographical distribution. For example, Indian isolates display a higher rate of resistance to CAS than those of other country (23.6% vs. 0.2%). In addition, within the United States, the level of resistance to AMB is reported at around 5% in the Midwest and West and 85% in the Mid-Atlantic [122]. To date, based on susceptibility profiles, echinocandins remain the first class of antifungals recommended to initiate chemotherapy. As mentioned lines above, *Candida* species can acquire or be naturally resistant to this class (or even more) of antifungals due to point mutations. Hence, there is urgency to develop novel, safe, and effective antifungals and treatment strategies with diverse drug targets to combat infections caused by resistant, as well as soon-to-be emerging fungal pathogens [122]. Notably, most genes associated with antifungal resistance are not directly involved in cell wall synthesis. This observation correlates with the relatively low resistance rates seen with echinocandins, which specifically target cell wall composition.

**Table 1:** List of genes and their mechanisms contributing to antifungal resistance.

Gene(s)	Function	Mutation or Mechanism	Reference
*FKS1*	Catalytic subunit of β-1,3-glucan synthase	Mutations K143R, Y132F, F126L	[122]
*CDR1* and *MDR1*	ABC transporter family (Multidrug efflux pump)	Point mutations or increased expression	[123]
* TAC1B *	Transcription factor controlling CDR1 expression	Mutation S611P	[124]
* MRR1A *	Transcriptional regulator of *MDR1*	Mutations V668G, Y813C	[123]
* ERG11 *	Encodes a lanosterol 14-a-demethylase	Point mutations or overexpression	[117]
* ERG1 *	Encodes a squalene epoxidase	Point mutations or overexpression	[117]
* ERG2 *	Encodes a C-8 sterol isomerase	Point mutations or overexpression	[117]
* ERG6 *	Encodes a D(24)-sterol C-methyltransferase	Point mutations or overexpression	[117]
*YPS1* and *YPS2*	Aspartic proteases	Overexpression	[125]
* SLT2 *	MAPK	Activation of CWI transcription factors	[126,127]

MAPK: mitogen-activated protein kinase; CWI: cell wall integrity.

Therefore, for new antifungal prospects and natural products offering alternative treatments for candidiasis (Table 2), the cell wall remains the primary target for these molecules [79]. Investigating the CWI-related signaling pathways, along with the regulatory mechanisms governing gene expression at both the upstream and downstream levels, could provide valuable insights for developing new antifungal agents or repurposing existing drugs to combat emerging resistant strains in clinical settings [64]. Some studies have shown the effects of deletion and expression inhibition on the cell wall and viability of *Candida* species, providing insights into biomolecules involved in CWI. For example, fatty acid elongases Fen1 and Fen2 may play a role in the CWI pathway, as *FEN1* and *FEN2* deletion mutants in *C. albicans* are more sensitive to cell wall stress [128]. The deletion of *SPF1* from *C. albicans* resulted in a strain sensitive to disruptive agents, yet resistant to CAS [129]. Also, the deletion of *HOG1* in *C. auris* leads to susceptibility to CAS [50]. On the other hand, *YPS1*, *YPK2*, and *SLT2* are involved in antifungal tolerance and CWI in *N. glabratus* [13]. Similarly, *MKC1* (*SLT2*) increases its expression upon CAS exposure [50]. Moreover, *N. glabratus* Erg6p has been proven to influence CWI and antifungal resistance [130]. New strategies for disrupting the function of these proteins may improve the treatment schemes.

Certain natural products like *O*-vanillin can disrupt CWI through diverse mechanisms. *O*-Vanillin alters the functional hydroxyl groups of the cell wall and reduces the amount of β-1,3-glucan in *Aspergillus flavus* [131]. The decreasing effect of vanillin on cell growth has been observed in *C. albicans* and other *Candida* species, including *N. glabratus* [132,133]. Also, vanillin-based fluoroindolines (DNA gyrase inhibitors) have shown antifungal effects in *C. albicans*; however, they require minimum inhibitory concentrations around 12–20 mg/mL [134]. The terpenes Citronellol, Pinene, and Menthone have shown promising antifungal effects on *C. albicans*, *N. glabratus*, and *C. tropicalis* [135]. Isospintanol, a terpene, inhibits biofilm formation, provokes mitochondrial dysfunction, and affects CWI, increasing chitin biosynthesis in *C. tropicalis* [136]. Moreover, the essential oils (EOs) from tea tree, cajeput, niaouli, and white thyme, which contain a high number of terpenes, were tested to assess their antifungal activity against *C. auris*. The EOs showed the inhibition of planktonic growth and biofilm formation [137]. The phenolic -OH group of terpenoids is known to penetrate and perturb fungal cell membranes; however, further research is needed to understand the mechanisms by which these compounds disrupt CWI [138]. In this way, Puupehenone, a Marine-Sponge-Derived sesquiterpene, interferes with the Hsp90 function in *S. cerevisiae* and potentiates the effectiveness of CAS [139]. Interestingly, geldanamycin, a benzoquinone inhibitor of Hsp90 ATPase activity, demonstrated enhanced antifungal effects against *C. auris* when used in combination with farnesol, a sesquiterpene. In contrast, combining geldanamycin with azoles, micafungin, or anidulafungin did not produce significant synergistic activity [113]. Similarly, geldanamycin in combination with fluconazole has shown positive effects against *C. albicans*, *N. glabratus*, and *C. parapsilosis*, but not against *C. auris* [140]. Although geldanamycin is a potent hepatotoxic compound, its structure may be useful to discover new drugs [141]. Radicicol is another Hsp90 inhibitor and also acts as an inhibitor of Ca^2+^ signaling [142]. Recently, the combination of radicicol with AMB, CAS, itraconazole, voriconazole, and fluconazole demonstrated effectiveness against *C. albicans*, *N. glabratus*, and *C. krusei* [143]. Similarly to geldanamycin, the uses of radicicol in drug design are currently under research [144]. Argifin was originally identified as a chitinase inhibitor produced by *Streptomyces* species [145]. In silico studies further suggest that argifin binds to 14α-lanosterol demethylase at the same target site as fluconazole [146]. However, due to its difficult synthesis and high-cost performance, argifin is currently used as a platform to design new chitinase inhibitors [147]. Phytolaccoside B produced by *Phytolacca tetramera* is a natural compound that has demonstrated antifungal effects against yeast [148]. Initially, this compound was associated with the inhibition of cell wall polymer synthesis and assembly [148]; nonetheless, it is currently known that Phytolaccoside B increases chitin synthase activity, arresting fungal growth. Extracts of *P. tetramera* in combination with azoles, AMB, and CAS showed growth and biofilm formation inhibition in multiresistant *C. albicans* [149].

Regarding new synthetic molecules, the compound 4-(3-(2,4-Dihydroxy-5-isopropylphenyl)-5-oxo-1,5-dihydro-4H-1,2,4-triazol-4-yl)-N-(8-(hydroxyamino)-8 oxooctyl) benzamide, known as J5, is a dual inhibitor of Hsp90 and histone deacetylase (HDAC) that has shown positive effects in combination with azoles in azole-resistant *C. albicans* [150]. Similar results have been demonstrated for the other inhibitors of HDAC, for example, novel BRD4 (bromodomain-containing protein 4)-HDAC dual inhibitors in combination with fluconazole. BRD4 is an epigenetic reader, which recognizes the acetylated lysine residues in histone tails, regulating gene expression [151]. These approximations remain under research. Nonetheless, the development of antifungals with the cell wall as a target is currently in progress. N-(4-Methoxyfumaroyl)-L-2,3-diaminopropanoic acid is an inhibitor of glucosamine-6-phosphate synthase [152]. This molecule has shown efficacy against *C. albicans*, including azole-resistant strains [153,154], and has also been effective in inhibiting *C. auris* growth [152]. PQA-AZ-13 is a new molecule based on indazole, pyrrolidine, and arylpiperazine with a trifluoromethyl moiety that showed positive activity against *C. auris* in vitro and ex vivo in a porcine skin model [155]. Fosmanogepix is a prodrug of manogepix that inhibits the acyltransferase enzyme Gwt1, which is essential for the maturation of GPI-anchored proteins. It has demonstrated in vivo activity against a broad range of pathogenic yeasts, including *C. albicans*, *N. glabratus*, and *C. auris* in a murine model of invasive candidiasis. This candidate has completed a Phase 2 clinical trial [156]. Rezafungin, a recently approved echinocandin for the treatment of candidemia and invasive candidiasis in both the United States and Europe, has demonstrated efficacy against several *Candida* species, including *C. albicans*, *C. auris*, *N. glabratus*, *C. dubliniensis*, *C. tropicalis*, and *C. parapsilosis* [127]. The mechanism of action of rezafungin is through the inhibition of glucan synthase [156]. Ibrezafungerp is an entumafungin-derived triterpenoid inhibitor of glucan synthase [157]. In vitro studies have shown its activity against *N. glabratus* isolates harboring *FKS* mutations [158] and its efficacy against fluconazole-resistant strains of *C. auris* and *C. albicans* [159]. Currently, ibrezafungerp is approved by the U.S. Food and Drug Administration [160] (Table 2).

**Table 2:** Prospects of antifungal molecules.

Compound	Target	Source	Mechanism of Action	Clinical Phase/Status	Reference
Ibrexafungerp	GS	Synthetic echinocandin	GS inhibitor	Approved by FDA	[160]
Rezafungin	GS	Synthetic echinocandin	GS inhibitor	Approved by FDA	[161]
Fosmanogepix	Gwt1	Synthetic prodrug of manogepix	Gwt1 inhibitor	Phase II	[162]
Radicicol	Chitin	* Monosporium bonorden *	Chs non-competitive inhibitor	-	[163]
Argifin	Chitin	*Streptomyces* spp.	Chitinase inhibitor	-	[145]
Phytolaccoside B	Chitin	* Phytolacca* *tetramera *	Chs activity enhancer	-	[164]
N-(4-Methoxyfumaroyl)-L-2,3-diaminopropanoic acid	GlcN-6-P synthase	Synthetic molecule	GlcN-6-P synthase inhibitor	-	[152]

GS: β-1,3-glucan synthase; Chs: chitin synthase; FDA: Food and Drug Administration.

**Figure 3:**
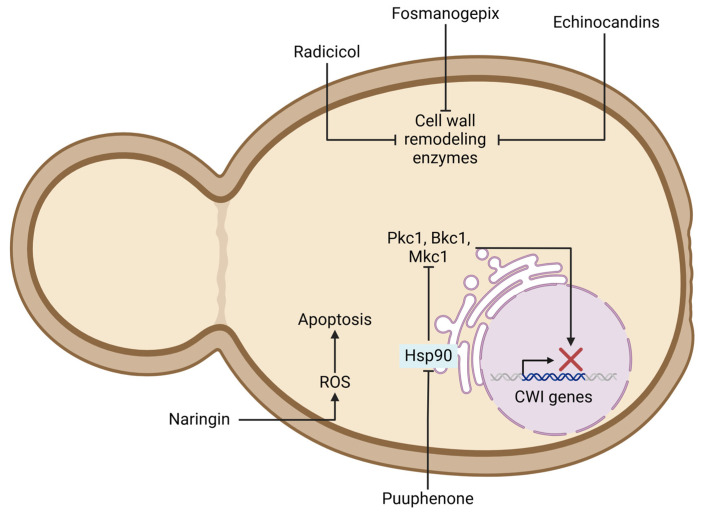
Potential antifungal strategies targeting fungal cell wall integrity (CWI) pathway and associated mechanisms. Figure highlights effects of compounds like echinocandins and fosmanogepix on cell-wall-remodeling enzymes, puupehenone-induced Hsp90 inhibition, and impact of naringin and on reactive oxygen species (ROS) generation and apoptosis.

Hence, developing new antifungal agents or repurposing existing drugs to target the signaling pathways that enable *Candida* species to adapt to stress appears to be a promising strategy for improving the management of candidiasis and candidemia. In addition, the increasing use of molecular docking tools can reveal previously unknown mechanisms of action, especially in natural compounds such as terpenes, which may affect CWI at multiple levels. This combined approach holds significant promise for identifying novel therapeutic strategies (Figure 3).

## 7. Future Directions

The previous studies have highlighted the significance of cell wall maintenance pathways, particularly the CWI pathway, in antifungal resistance, virulence, and cell viability. The emergence of multiresistant *Candida* species requires the improvement or discovery of new therapeutic strategies to extend the arsenal of antifungals, and then alleviate complications in treatment due to multiresistant strains or species of *Candida*. Also, diagnosis remains a challenging field. The phenotypic variations within *C. auris* species occurring in culture media can complicate identification. Research on the development of specific biomarkers may optimize diagnosis. Overall, new research should be focused on the impact of downstream effectors and chaperone proteins, as well as on gene regulation at transcriptional, post-transcriptional, and translational levels. One promising direction is the structural study of proteins involved in the CWI response, which could deepen our understanding of their roles in pathogenicity and resistance mechanisms. Additionally, advances in artificial intelligence and molecular docking offer opportunities for the design and prediction of novel molecules that optimize or provide alternative therapeutic options for candidiasis, particularly in systemic infections. The new approaches may include genomic strategies to develop antifungal agents, leveraging the wealth of data from fungal genomes to identify novel drug targets.

Furthermore, combinatorial treatments, for example chaperone inhibitors with antifungals and agents that interfere with cell wall biosynthesis, have shown potential to improve clinical outcomes, highlighting the importance of drug synergy in antifungal therapy. Targeting the downstream effectors or regulatory proteins of the cell wall maintenance pathways may be useful to successfully developing new therapeutic approaches to treating candidiasis caused by multidrug-resistant strains.

## Data Availability

No new data were created or analyzed in this study.

## References

[1] Bhattacharya S., Sae-Tia S., Fries B.C. (2020). Candidiasis and Mechanisms of Antifungal Resistance. Antibiotics.

[2] Barantsevich N., Barantsevich E. (2022). Diagnosis and Treatment of Invasive Candidiasis. Antibiotics.

[3] Thomas-Rüddel D.O., Schlattmann P., Pletz M., Kurzai O., Bloos F. (2022). Risk Factors for Invasive Candida Infection in Critically Ill Patients: A Systematic Review and Meta-Analysis. Chest.

[4] Stenkiewicz-Witeska J.S., Ene I.V. (2023). Azole Potentiation in Candida Species. PLoS Pathog..

[5] Mroczyńska M., Brillowska-Dąbrowska A. (2020). Review on Current Status of Echinocandins Use. Antibiotics.

[6] Logan C., Martin-Loeches I., Bicanic T. (2020). Invasive Candidiasis in Critical Care: Challenges and Future Directions. Intensive Care Med..

[7] Frías-De-León M.G., Hernández-Castro R., Vite-Garín T., Arenas R., Bonifaz A., Castañón-Olivares L., Acosta-Altamirano G., Martínez-Herrera E. (2020). Antifungal Resistance in Candida Auris: Molecular Determinants. Antibiotics.

[8] Czajka K.M., Venkataraman K., Brabant-Kirwan D., Santi S.A., Verschoor C., Appanna V.D., Singh R., Saunders D.P., Tharmalingam S. (2023). Molecular Mechanisms Associated with Antifungal Resistance in Pathogenic Candida Species. Cells.

[9] Paul S., Shaw D., Joshi H., Singh S., Chakrabarti A., Rudramurthy S.M., Ghosh A.K. (2022). Mechanisms of Azole Antifungal Resistance in Clinical Isolates of *Candida tropicalis*. PLoS ONE.

[10] Milholland K.L., AbdelKhalek A., Baker K.M., Hoda S., DeMarco A.G., Naughton N.H., Koeberlein A.N., Lorenz G.R., Anandasothy K., Esperilla-Muñoz A. (2022). Reduced Cdc14 Phosphatase Activity Impairs Septation, Hyphal Differentiation and Pathogenesis and Causes Echinocandin Hypersensitivity in *Candida albicans*. bioRxiv.

[11] Lenardon M.D., Sood P., Dorfmueller H.C., Brown A.J.P., Gow N.A.R. (2020). Scalar Nanostructure of the Candida Albicans Cell Wall; a Molecular, Cellular and Ultrastructural Analysis and Interpretation. Cell Surf..

[12] Ahmadipour S., Field R.A., Miller G.J. (2021). Prospects for Anti-Candida Therapy through Targeting the Cell Wall: A Mini-Review. Cell Surf..

[13] Garcia-Rubio R., Hernandez R.Y., Clear A., Healey K.R., Shor E., Perlin D.S. (2021). Critical Assessment of Cell Wall Integrity Factors Contributing to in Vivo Echinocandin Tolerance and Resistance in *Candida glabrata*. Front. Microbiol..

[14] Pérez P., Cortés J.C.G., Cansado J., Ribas J.C. (2018). Fission Yeast Cell Wall Biosynthesis and Cell Integrity Signalling. Cell Surf..

[15] Hopke A., Brown A.J.P., Hall R.A., Wheeler R.T. (2018). Dynamic Fungal Cell Wall Architecture in Stress Adaptation and Immune Evasion. Trends Microbiol..

[16] Stewart G.G., Stewart G.G. (2017). The Structure and Function of the Yeast Cell Wall, Plasma Membrane and Periplasm. Brewing and Distilling Yeasts.

[17] Pérez-García L.A., Csonka K., Flores-Carreón A., Estrada-Mata E., Mellado-Mojica E., Németh T., López-Ramírez L.A., Toth R., López M.G., Vizler C. (2016). Role of Protein Glycosylation in *Candida parapsilosis* Cell Wall Integrity and Host Interaction. Front. Microbiol..

[18] Ribeiro R.A., Bourbon-Melo N., Sá-Correia I. (2022). The Cell Wall and the Response and Tolerance to Stresses of Biotechnological Relevance in Yeasts. Front. Microbiol..

[19] Bemena L.D., Min K., Konopka J.B., Neiman A.M. (2021). A Conserved Machinery Underlies the Synthesis of a Chitosan Layer in the Candida Chlamydospore Cell Wall. mSphere.

[20] Schild L., Heyken A., de Groot P.W.J., Hiller E., Mock M., de Koster C., Horn U., Rupp S., Hube B. (2011). Proteolytic Cleavage of Covalently Linked Cell Wall Proteins by Candida Albicans Sap9 and Sap10. Eukaryot. Cell.

[21] Brown H.E., Esher S.K., Alspaugh J.A. (2020). Chitin: A “Hidden Figure” in the Fungal Cell Wall. Curr. Top. Microbiol. Immunol..

[22] Garcia-Rubio R., de Oliveira H.C., Rivera J., Trevijano-Contador N. (2020). The Fungal Cell Wall: Candida, Cryptococcus, and Aspergillus Species. Front. Microbiol..

[23] Lowman D.W., Sameer Al-Abdul-Wahid M., Ma Z., Kruppa M.D., Rustchenko E., Williams D.L. (2021). Glucan and Glycogen Exist as a Covalently Linked Macromolecular Complex in the Cell Wall of Candida Albicans and Other Candida Species. Cell Surf..

[24] de Assis L.J., Bain J.M., Liddle C., Leaves I., Hacker C., da Silva R.P., Yuecel R., Bebes A., Stead D., Childers D.S. (2022). Nature of B-1,3-Glucan-Exposing Features on Candida Albicans Cell Wall and Their Modulation. mBio.

[25] Wang Y., Zou Y., Chen X., Li H., Yin Z., Zhang B., Xu Y., Zhang Y., Zhang R., Huang X. (2022). Innate Immune Responses against the Fungal Pathogen Candida Auris. Nat. Commun..

[26] Widanage M.C.D., Singh K., Li J., Yarava J.R., Scott F.J., Xu Y., Gow N.A.R., Mentink-Vigier F., Wang P., Lamoth F. (2024). Unveiling Cell Wall Structure and Echinocandin Response in Candida Auris and Candida Albicans via Solid-State NMR. bioRxiv.

[27] Navarro-Arias M.J., Hernández-Chávez M.J., Garcia-Carnero L.C., Amezcua-Hernández D.G., Lozoya-Pérez N.E., Estrada-Mata E., Martínez-Duncker I., Franco B., Mora-Montes H.M. (2019). Differential Recognition of *Candida tropicalis*, Candida Guilliermondii, Candida Krusei, and Candida Auris by Human Innate Immune Cells. Infect. Drug Resist..

[28] Takahashi S., Kudoh A., Okawa Y., Shibata N. (2012). Significant Differences in the Cell-Wall Mannans from Three *Candida glabrata* Strains Correlate with Antifungal Drug Sensitivity. FEBS J..

[29] Vitali A., Vavala E., Marzano V., Leone C., Castagnola M., Iavarone F., Angiolella L. (2017). Cell Wall Composition and Biofilm Formation of Azoles-Susceptible and -Resistant *Candida glabrata* Strains. J. Chemother..

[30] Costa-de-Oliveira S., Silva A.P., Miranda I.M., Salvador A., Azevedo M.M., Munro C.A., Rodrigues A.G., Pina-Vaz C. (2013). Determination of Chitin Content in Fungal Cell Wall: An Alternative Flow Cytometric Method. Cytom. Part A.

[31] Plaine A., Walker L., Da Costa G., Mora-Montes H.M., McKinnon A., Gow N.A.R., Gaillardin C., Munro C.A., Richard M.L. (2008). Functional Analysis of Candida Albicans GPI-Anchored Proteins: Roles in Cell Wall Integrity and Caspofungin Sensitivity. Fungal. Genet. Biol..

[32] Alvarado M., Gómez-Navajas J.A., Blázquez-Muñoz M.T., Gómez-Molero E., Fernández-Sánchez S., Eraso E., Munro C.A., Valentín E., Mateo E., de Groot P.W.J. (2024). The Good, the Bad, and the Hazardous: Comparative Genomic Analysis Unveils Cell Wall Features in the Pathogen Candidozyma Auris Typical for Both Baker’s Yeast and Candida. FEMS Yeast Res..

[33] Duggan S., Usher J. (2023). *Candida glabrata*: A Powerhouse of Resistance. PLoS Pathog..

[34] Zhang Y., Gong S., Xiong K., Yu X., Mo X., Su C., Lu Y. (2024). An Alteration in the Expression of Cell Wall Structural Proteins Increases Cell Surface Exposure of Adhesins to Promote Virulence in *Candida glabrata*. mSphere.

[35] Kim J., Oh S.H., Rodriguez-Bobadilla R., Vuong V.M., Hubka V., Zhao X., Hoyer L.L. (2022). Peering Into Candida Albicans Pir Protein Function and Comparative Genomics of the Pir Family. Front. Cell Infect. Microbiol..

[36] De Groot P.W.J., De Boer A.D., Cunningham J., Dekker H.L., De Jong L., Hellingwerf K.J., De Koster C., Klis F.M. (2004). Proteomic Analysis of Candida Albicans Cell Walls Reveals Covalently Bound Carbohydrate-Active Enzymes and Adhesins. Eukaryot. Cell.

[37] Ceballos-Garzon A., Monteoliva L., Gil C., Alvarez-Moreno C., Vega-Vela N.E., Engelthaler D.M., Bowers J., Le Pape P., Parra-Giraldo C.M. (2022). Genotypic, Proteomic, and Phenotypic Approaches to Decipher the Response to Caspofungin and Calcineurin Inhibitors in Clinical Isolates of Echinocandin-Resistant *Candida glabrata*. J. Antimicrob. Chemother..

[38] Satala D., Karkowska-Kuleta J., Zelazna A., Rapala-Kozik M., Kozik A. (2020). Moonlighting Proteins at the Candidal Cell Surface. Microorganisms.

[39] Arvizu-Rubio V.J., García-Carnero L.C., Mora-Montes H.M. (2022). Moonlighting Proteins in Medically Relevant Fungi. PeerJ.

[40] Urban C., Xiong X., Sohn K., Schröppel K., Brunner H., Rupp S. (2005). The Moonlighting Protein Tsa1p Is Implicated in Oxidative Stress Response and in Cell Wall Biogenesis in Candida Albicans. Mol. Microbiol..

[41] Paudyal A., Vediyappan G. (2021). Cell Surface Expression of Nrg1 Protein in Candida Auris. J. Fungi.

[42] Karkowska-Kuleta J., Satala D., Bochenska O., Rapala-Kozik M., Kozik A. (2019). Moonlighting Proteins Are Variably Exposed at the Cell Surfaces of *Candida glabrata*, *Candida parapsilosis* and *Candida tropicalis* under Certain Growth Conditions. BMC Microbiol..

[43] Gow N.A.R., Latge J.-P., Munro C.A. (2017). The Fungal Cell Wall: Structure, Biosynthesis, and Function. Microbiol. Spectr..

[44] Liu N.N., Acosta-Zaldívar M., Qi W., Diray-Arce J., Walker L.A., Kottom T.J., Kelly R., Yuan M., Asara J.M., Lasky-Su J.A. (2020). Phosphoric Metabolites Link Phosphate Import and Polysaccharide Biosynthesis for Candida Albicans Cell Wall Maintenance. mBio.

[45] Allert S., Schulz D., Kämmer P., Großmann P., Wolf T., Schäuble S., Panagiotou G., Brunke S., Hube B. (2022). From Environmental Adaptation to Host Survival: Attributes That Mediate Pathogenicity of Candida Auris. Virulence.

[46] Köhler J.R., Acosta-Zaldívar M., Qi W. (2020). Phosphate in Virulence of Candida Albicans and *Candida glabrata*. J. Fungi.

[47] Ibe C., Munro C.A. (2021). Fungal Cell Wall: An Underexploited Target for Antifungal Therapies. PLoS Pathog..

[48] Razmi M., Kim J., Chinnici J., Busarajan S., Vuppalapaty H., Lankipalli D., Li R., Maddi A. (2024). Candida Albicans Mannosidases, Dfg5 and Dcw1, Are Required for Cell Wall Integrity and Pathogenesis. J. Fungi.

[49] Pavesic M.W., Gale A.N., Nickels T.J., Harrington A.A., Bussey M., Cunningham K.W. (2024). Calcineurin-Dependent Contributions to Fitness in the Opportunistic Pathogen *Candida glabrata*. mSphere.

[50] Zamith-Miranda D., Amatuzzi R.F., Munhoz da Rocha I.F., Martins S.T., Lucena A.C.R., Vieira A.Z., Trentin G., Almeida F., Rodrigues M.L., Nakayasu E.S. (2021). Transcriptional and Translational Landscape of Candida Auris in Response to Caspofungin. Comput. Struct. Biotechnol. J..

[51] Jain P., Sethi S.C., Pratyusha V.A., Garai P., Naqvi N., Singh S., Pawar K., Puri N., Komath S.S. (2018). Ras Signaling Activates Glycosylphosphatidylinositol (GPI) Anchor Biosynthesis via the GPI–N-Acetylglucosaminyltransferase (GPI–GnT) in Candida Albicans. J. Biol. Chem..

[52] de Oliveira H.C., Rossi S.A., García-Barbazán I., Zaragoza Ó., Trevijano-Contador N. (2021). Cell Wall Integrity Pathway Involved in Morphogenesis, Virulence and Antifungal Susceptibility in Cryptococcus Neoformans. J. Fungi.

[53] Jiménez-Gutiérrez E., Alegría-Carrasco E., Sellers-Moya Á., Molina M., Martín H. (2020). Not Just the Wall: The Other Ways to Turn the Yeast CWI Pathway On. Int. Microbiol..

[54] Ibe C., Munro C.A. (2021). Fungal Cell Wall Proteins and Signaling Pathways Form a Cytoprotective Network to Combat Stresses. J. Fungi.

[55] Sanz A.B., Díez-Muñiz S., Moya J., Petryk Y., Nombela C., Rodríguez-Peña J.M., Arroyo J. (2022). Systematic Identification of Essential Genes Required for Yeast Cell Wall Integrity: Involvement of the RSC Remodelling Complex. J. Fungi.

[56] Wang W.-H., Lai T.-X., Wu Y.-C., Chen Z.-T., Tseng K.-Y., Lan C.-Y. (2022). Associations of Rap1 with Cell Wall Integrity, Biofilm Formation, and Virulence in Candida Albicans. Microbiol. Spectr..

[57] Yeh Y.-C., Wang H.-Y., Lan C.-Y. (2020). Candida Albicans Aro1 Affects Cell Wall Integrity, Biofilm Formation and Virulence. J. Microbiol. Immunol. Infect..

[58] Shivarathri R., Jenull S., Stoiber A., Chauhan M., Mazumdar R., Singh A., Nogueira F., Kuchler K., Chowdhary A., Chauhan N. (2020). The Two-Component Response Regulator Ssk1 and the Mitogen-Activated Protein Kinase Hog1 Control Antifungal Drug Resistance and Cell Wall Architecture of Candida Auris. mSphere.

[59] Heredia M.Y., Ikeh M.A.C., Gunasekaran D., Conrad K.A., Filimonava S., Marotta D.H., Nobile C.J., Rauceo J.M. (2020). An Expanded Cell Wall Damage Signaling Network Is Comprised of the Transcription Factors Rlm1 and Sko1 in Candida Albicans. PLoS Genet..

[60] Kim J.-S., Lee K.-T., Lee M.H., Cheong E., Bahn Y.-S. (2021). Adenylyl Cyclase and Protein Kinase A Play Redundant and Distinct Roles in Growth, Differentiation, Antifungal Drug Resistance, and Pathogenicity of Candida Auris. mBio.

[61] González-Rubio G., Sastre-Vergara L., Molina M., Martín H., Fernández-Acero T. (2022). Substrates of the MAPK Slt2: Shaping Yeast Cell Integrity. J. Fungi.

[62] Sanz A.B., García R., Rodríguez-Peña J.M., Arroyo J. (2018). The CWI Pathway: Regulation of the Transcriptional Adaptive Response to Cell Wall Stress in Yeast. J. Fungi.

[63] Balachandra V.K., Verma J., Shankar M., Tucey T.M., Traven A., Schittenhelm R.B., Ghosh S.K. (2020). The RSC (Remodels the Structure of Chromatin) Complex of Candida Albicans Shows Compositional Divergence with Distinct Roles in Regulating Pathogenic Traits. PLoS Genet..

[64] Sanz A.B., García R., Pavón-Vergés M., Rodríguez-Peña J.M., Arroyo J. (2022). Control of Gene Expression via the Yeast CWI Pathway. Int. J. Mol. Sci..

[65] Wang L., Chen R., Weng Q., Lin S., Wang H., Li L., Fuchs B.B., Tan X., Mylonakis E. (2020). SPT20 Regulates the Hog1-MAPK Pathway and Is Involved in Candida Albicans Response to Hyperosmotic Stress. Front. Microbiol..

[66] Dong Y., Du J., Deng Y., Cheng M., Shi Z., Zhu H., Sun H., Yu Q., Li M. (2024). Reduction of Histone Proteins Dosages Increases CFW Sensitivity and Attenuates Virulence of Candida Albicans. Microbiol. Res..

[67] Zhang L., Liu N., Ma X., Jiang L. (2013). The Transcriptional Control Machinery as Well as the Cell Wall Integrity and Its Regulation Are Involved in the Detoxification of the Organic Solvent Dimethyl Sulfoxide in Saccharomyces Cerevisiae. FEMS Yeast Res..

[68] Viéitez C., Martínez-Cebrián G., Solé C., Böttcher R., Potel C.M., Savitski M.M., Onnebo S., Fabregat M., Shilatifard A., Posas F. (2020). A Genetic Analysis Reveals Novel Histone Residues Required for Transcriptional Reprogramming upon Stress. Nucleic Acids Res..

[69] Chen C., Zhang Y., Zeng L., HUANG X., Wang Y., Chen G., Moses M., Zou Y., Xiong S., Xue W. (2024). Targeting Epigenetic Regulators to Overcome Drug Resistance in the Emerging Human Fungal Pathogen Candida Auris. preprint.

[70] Kumar K., Pareek A., Kaur R. (2024). SWI/SNF Complex-Mediated Chromatin Remodeling in *Candida glabrata* Promotes Immune Evasion. iScience.

[71] Filler E.E., Liu Y., Solis N.V., Wang L., Diaz L.F., Edwards J.E., Filler S.G., Yeaman M.R. (2021). Identification of *Candida glabrata* Transcriptional Regulators That Govern Stress Resistance and Virulence. Infect. Immun..

[72] Su S., Li X., Yang X., Li Y., Chen X., Sun S., Jia S. (2020). Histone Acetylation/Deacetylation in Candida Albicans and Their Potential as Antifungal Targets. Future Microbiol..

[73] Bresson S., Shchepachev V., Tollervey D. (2023). A Posttranscriptional Pathway Regulates Cell Wall MRNA Expression in Budding Yeast. Cell Rep..

[74] Jnied M.M. (2024). Post-Transcriptional Regulation of Fungal Cell Walls by RNA-Binding Proteins and Untranslated Regions. Ph.D. Thesis.

[75] Healey K.R., Paderu P., Hou X., Jimenez Ortigosa C., Bagley N., Patel B., Zhao Y., Perlin D.S. (2020). Differential Regulation of Echinocandin Targets Fks1 and Fks2 in *Candida glabrata* by the Post-Transcriptional Regulator Ssd1. J. Fungi.

[76] Hall R.A., Wallace E.W.J. (2022). Post-Transcriptional Control of Fungal Cell Wall Synthesis. Cell Surf..

[77] Cañonero L., Pautasso C., Galello F., Sigaut L., Pietrasanta L., Arroyo J., Bermúdez-Moretti M., Portela P., Rossi S. (2022). Heat Stress Regulates the Expression of TPK1 Gene at Transcriptional and Post-Transcriptional Levels in Saccharomyces Cerevisiae. Biochim. et Biophys. Acta (BBA)-Mol. Cell Res..

[78] Liu J., Zhang W., Long S., Zhao C. (2021). Maintenance of Cell Wall Integrity under High Salinity. Int. J. Mol. Sci..

[79] Liu W., Yuan L., Wang S. (2020). Recent Progress in the Discovery of Antifungal Agents Targeting the Cell Wall. J. Med. Chem..

[80] Iyer K.R., Robbins N., Cowen L.E. (2022). The Role of Candida Albicans Stress Response Pathways in Antifungal Tolerance and Resistance. iScience.

[81] Gerik K.J., Donlin M.J., Soto C.E., Banks A.M., Banks I.R., Maligie M.A., Selitrennikoff C.P., Lodge J.K. (2005). Cell Wall Integrity Is Dependent on the PKC1 Signal Transduction Pathway in Cryptococcus Neoformans. Mol. Microbiol..

[82] Alonso-Monge R., Guirao-Abad J.P., Sánchez-Fresneda R., Pla J., Yagüe G., Argüelles J.C. (2020). The Fungicidal Action of Micafungin Is Independent on Both Oxidative Stress Generation and HOG Pathway Signaling in Candida Albicans. Microorganisms.

[83] García R., Itto-Nakama K., Rodríguez-Peña J.M., Chen X., Sanz A.B., de Lorenzo A., Pavón-Vergés M., Kubo K., Ohnuki S., Nombela C. (2021). Poacic Acid, a β-1,3-Glucan–Binding Antifungal Agent, Inhibits Cell-Wall Remodeling and Activates Transcriptional Responses Regulated by the Cell-Wall Integrity and High-Osmolarity Glycerol Pathways in Yeast. FASEB J..

[84] Blomberg A. (2022). Yeast Osmoregulation–Glycerol Still in Pole Position. FEMS Yeast Res..

[85] de Nadal E., Posas F. (2022). The HOG Pathway and the Regulation of Osmoadaptive Responses in Yeast. FEMS Yeast Res..

[86] Duveau F., Cordier C., Chiron L., Le Bec M., Pouzet S., Séguin J., Llamosi A., Sorre B., Di Meglio J.-M., Hersen P. (2024). Yeast Cell Responses and Survival during Periodic Osmotic Stress Are Controlled by Glucose Availability. Elife.

[87] Saldaña C., Villava C., Ramírez-Villarreal J., Morales-Tlalpan V., Campos-Guillen J., Chávez-Servín J., García-Gasca T. (2021). Rapid and Reversible Cell Volume Changes in Response to Osmotic Stress in Yeast. Braz. J. Microbiol..

[88] Herrero-de-Dios C., Román E., Pla J., Alonso-Monge R. (2020). Hog1 Controls Lipids Homeostasis Upon Osmotic Stress in Candida Albicans. J. Fungi.

[89] Huang Y.-M., Tao X.-H., Xu D.-F., Yu Y., Teng Y., Xie W.-Q., Fan Y.-B. (2021). HOG1 Has an Essential Role in the Stress Response, Virulence and Pathogenicity of EmCryptococcus Gattii/Em. Exp. Ther. Med..

[90] Guirao-Abad J.P., Sánchez-Fresneda R., Román E., Pla J., Argüelles J.C., Alonso-Monge R. (2020). The MAPK Hog1 Mediates the Response to Amphotericin B in Candida Albicans. Fungal Genet. Biol..

[91] Sahu M.S., Purushotham R., Kaur R. (2024). The Hog1 MAPK Substrate Governs *Candida glabrata*-Epithelial Cell Adhesion via the Histone H2A Variant. PLoS Genet..

[92] Cui S., Li M., Hassan R.Y.A., Heintz-Buschart A., Wang J., Bilitewski U. (2020). Inhibition of Respiration of Candida Albicans by Small Molecules Increases Phagocytosis Efficacy by Macrophages. mSphere.

[93] Kim H., Lee D.G. (2021). Naringin-Generated ROS Promotes Mitochondria-Mediated Apoptosis in *Candida albicans*. IUBMB Life.

[94] Ferrari S., Sanguinetti M., De Bernardis F., Torelli R., Posteraro B., Vandeputte P., Sanglard D. (2011). Loss of Mitochondrial Functions Associated with Azole Resistance in *Candida glabrata* Results in Enhanced Virulence in Mice. Antimicrob. Agents Chemother..

[95] Simm C., Weerasinghe H., Thomas D.R., Harrison P.F., Newton H.J., Beilharz T.H., Traven A. (2022). Disruption of Iron Homeostasis and Mitochondrial Metabolism Are Promising Targets to Inhibit Candida Auris. Microbiol. Spectr..

[96] Chen H., Zhou X., Ren B., Cheng L. (2020). The Regulation of Hyphae Growth in Candida Albicans. Virulence.

[97] Neeli-Venkata R., Diaz C.M., Celador R., Sanchez Y., Minc N. (2021). Detection of Surface Forces by the Cell-Wall Mechanosensor Wsc1 in Yeast. Dev. Cell.

[98] Lodder A.L., Lee T.K., Ballester R. (1999). Characterization of the Wsc1 Protein, a Putative Receptor in the Stress Response of Saccharomyces Cerevisiae. Genetics.

[99] Puerner C., Kukhaleishvili N., Thomson D., Schaub S., Noblin X., Seminara A., Bassilana M., Arkowitz R.A. (2020). Mechanical Force-Induced Morphology Changes in a Human Fungal Pathogen. BMC Biol..

[100] Milne G., Walker L.A. (2022). High-Pressure Freezing and Transmission Electron Microscopy to Visualize the Ultrastructure of the C. Auris Cell Wall. Methods Mol. Biol..

[101] Atamas A.A., Guskov A.I., Rogachev A.V. (2023). Structural Study of the Candida Auris Ribosome. Mosc. Univ. Biol. Sci. Bull..

[102] Jiang J., Keniya M.V., Puri A., Zhan X., Cheng J., Wang H., Lin G., Lee Y.-K., Jaber N., Hassoun Y. (2024). Structural and Biophysical Dynamics of Fungal Plasma Membrane Proteins and Implications for Echinocandin Action in *Candida glabrata*. bioRxiv.

[103] Jiménez-Ortigosa C., Jiang J., Chen M., Kuang X., Healey K.R., Castellano P., Boparai N., Ludtke S.J., Perlin D.S., Dai W. (2021). Cryo-Electron Tomography of *Candida glabrata* Plasma Membrane Proteins. J. Fungi.

[104] Jiang J., Jiménez-Ortigosa C., Chen M., Healey K.R., Kong J., Lee Y.-K., Farrell D.P., DiMaio F., Perlin D.S., Dai W. (2022). Elucidating the 3D Structure of β-(1,3)-Glucan Synthase from *Candida glabrata* by Subtomogram Averaging. Microsc. Microanal..

[105] Turk M., Baumeister W. (2020). The Promise and the Challenges of Cryo-Electron Tomography. FEBS Lett..

[106] Petrokilidou C., Pavlou E., Velegraki A., Simou A., Marsellou I., Filis G., Bassukas I.D., Gaitanis G., Kourkoumelis N. (2024). Characterization and Differentiation of Candida Auris on Dixon’s Agar Using Raman Spectroscopy. Pathogens.

[107] Kudla G., Wan Y., Helwak A. (2020). RNA Conformation Capture by Proximity Ligation. Annu. Rev. Genom. Hum. Genet..

[108] Bentsen M., Goymann P., Schultheis H., Klee K., Petrova A., Wiegandt R., Fust A., Preussner J., Kuenne C., Braun T. (2020). ATAC-Seq Footprinting Unravels Kinetics of Transcription Factor Binding during Zygotic Genome Activation. Nat. Commun..

[109] Liao B., Ye X., Chen X., Zhou Y., Cheng L., Zhou X., Ren B. (2021). The Two-Component Signal Transduction System and Its Regulation in Candida Albicans. Virulence.

[110] Kotey F.C., Dayie N.T., Tetteh-Uarcoo P.B., Donkor E.S. (2021). Candida Bloodstream Infections: Changes in Epidemiology and Increase in Drug Resistance. Infect. Dis. Res. Treat..

[111] Parslow B.Y., Thornton C.R. (2022). Continuing Shifts in Epidemiology and Antifungal Susceptibility Highlight the Need for Improved Disease Management of Invasive Candidiasis. Microorganisms.

[112] Perlin D.S. (2020). Cell Wall-Modifying Antifungal Drugs. Curr. Top. Microbiol. Immunol..

[113] Cândido E.d.S., Affonseca F., Cardoso M.H., Franco O.L. (2020). Echinocandins as Biotechnological Tools for Treating Candida Auris Infections. J. Fungi.

[114] Liu F., Zhong L., Zhou F., Zheng C., Zhang K., Cai J., Zhou H., Tang K., Dong Z., Cui W. (2021). Clinical Features, Strain Distribution, Antifungal Resistance and Prognosis of Patients with Non-Albicans Candidemia: A Retrospective Observational Study. Infect. Drug Resist..

[115] Bohner F., Papp C., Gácser A. (2022). The Effect of Antifungal Resistance Development on the Virulence of Candida Species. FEMS Yeast Res..

[116] Ben-Ami R., Kontoyiannis D.P. (2021). Resistance to Antifungal Drugs. Infect. Dis. Clin..

[117] Sharma C., Kadosh D. (2023). Perspective on the Origin, Resistance, and Spread of the Emerging Human Fungal Pathogen Candida Auris. PLoS Pathog..

[118] Izumi H., Nafie L.A., Dukor R.K. (2024). Effect of Conformational Variability on the Drug Resistance of Candida Auris ERG11p and FKS1. ACS Omega.

[119] Frías-De-león M.G., Hernández-Castro R., Conde-Cuevas E., García-Coronel I.H., Vázquez-Aceituno V.A., Soriano-Ursúa M.A., Farfán-García E.D., Ocharán-Hernández E., Rodríguez-Cerdeira C., Arenas R. (2021). *Candida glabrata* Antifungal Resistance and Virulence Factors, a Perfect Pathogenic Combination. Pharmaceutics.

[120] Badrane H., Cheng S., Dupont C.L., Hao B., Driscoll E., Morder K., Liu G., Newbrough A., Fleres G., Kaul D. (2023). Genotypic Diversity and Unrecognized Antifungal Resistance among Populations of *Candida glabrata* from Positive Blood Cultures. Nat. Commun..

[121] Kappel D., Gifford H., Brackin A., Abdolrasouli A., Eyre D.W., Jeffery K., Schlenz S., Aanensen D.M., Brown C.S., Borman A. (2024). Genomic Epidemiology Describes Introduction and Outbreaks of Antifungal Drug-Resistant Candida Auris. Npj Antimicrob. Resist..

[122] Eix E.F., Nett J.E. (2025). *Candida auris*: Epidemiology and Antifungal Strategy. Annu. Rev. Med..

[123] Borgeat V., Brandalise D., Grenouillet F., Sanglard D. (2021). Participation of the Abc Transporter Cdr1 in Azole Resistance of Candida Lusitaniae. J. Fungi.

[124] Li J., Coste A.T., Liechti M., Bachmann D., Sanglard D., Lamoth F. (2021). Novel ERG11 and TAC1b Mutations Associated with Azole Resistance in Candida Auris. Antimicrob. Agents Chemother..

[125] Bednarek A., Kabut A., Rapala-Kozik M., Satala D. (2024). Exploring the Effects of Culture Conditions on Yapsin (YPS) Gene Expression in Nakaseomyces Glabratus. Open Life Sci..

[126] Nagayoshi Y., Miyazaki T., Minematsu A., Yamauchi S., Takazono T., Nakamura S., Imamura Y., Izumikawa K., Kakeya H., Yanagihara K. (2014). Contribution of the Slt2-Regulated Transcription Factors to Echinocandin Tolerance in *Candida glabrata*. FEMS Yeast Res..

[127] Reinoso-Martín C., Schüller C., Schuetzer-Muehlbauer M., Kuchler K. (2003). The Yeast Protein Kinase C Cell Integrity Pathway Mediates Tolerance to the Antifungal Drug Caspofungin through Activation of Slt2p Mitogen-Activated Protein Kinase Signaling. Eukaryot. Cell.

[128] Song J., Liu X., Li R. (2020). Sphingolipids: Regulators of Azole Drug Resistance and Fungal Pathogenicity. Mol. Microbiol..

[129] Chang C.K., Yang M.C., Chen H.F., Liao Y.L., Lan C.Y. (2022). The Role of Sfp1 in Candida Albicans Cell Wall Maintenance. J. Fungi.

[130] Elias D., Toth Hervay N., Jacko J., Morvova M., Valachovic M., Gbelska Y. (2022). Erg6p Is Essential for Antifungal Drug Resistance, Plasma Membrane Properties and Cell Wall Integrity in *Candida glabrata*. FEMS Yeast Res..

[131] Li Q., Zhu X., Xie Y., Zhong Y. (2021). O-Vanillin, a Promising Antifungal Agent, Inhibits Aspergillus Flavus by Disrupting the Integrity of Cell Walls and Cell Membranes. Appl. Microbiol. Biotechnol..

[132] Mikulášová M., Vodný Š., Pekarovičová A. (1990). Influence of Phenolics on Biomass Production by Candida Utilis and Candida Albicans. Biomass.

[133] Sri P.V.L., Srinivasan S., Muthukumar S., Chellaswamy S., Nachiappan N.N., Thamilselvan S. (2023). Evaluation of Antifungal Activity of Vanilla Pods Silver Nanoparticles against Various Oral Candidal Species: An In-vitro Study. J. Oral Maxillofac. Pathol..

[134] Raymond Mohanraj D.G., Alagumuthu M., Subramaniam P., Bakthavachalam D., Arumugam S., Chellam S. (2021). Antimicrobial Effects of Vanillin-Based Pyridyl-Benzylidene-5-Fluoroindolins. J. Heterocycl. Chem..

[135] Iraji A., Yazdanpanah S., Alizadeh F., Mirzamohammadi S., Ghasemi Y., Pakshir K., Yang Y., Zomorodian K. (2020). Screening the Antifungal Activities of Monoterpenes and Their Isomers against Candida Species. J. Appl. Microbiol..

[136] Contreras Martínez O.I., Angulo Ortíz A., Santafé Patiño G., Peñata-Taborda A., Berrio Soto R. (2023). Isoespintanol Antifungal Activity Involves Mitochondrial Dysfunction, Inhibition of Biofilm Formation, and Damage to Cell Wall Integrity in *Candida tropicalis*. Int. J. Mol. Sci..

[137] Fernandes L., Ribeiro R., Costa R., Henriques M., Rodrigues M.E. (2022). Essential Oils as a Good Weapon against Drug-Resistant Candida Auris. Antibiotics.

[138] Konuk H.B., Ergüden B. (2020). Phenolic –OH Group Is Crucial for the Antifungal Activity of Terpenoids via Disruption of Cell Membrane Integrity. Folia Microbiol..

[139] Tripathi S.K., Feng Q., Liu L., Levin D.E., Roy K.K., Doerksen R.J., Baerson S.R., Shi X., Pan X., Xu W.-H. (2020). Puupehenone, a Marine-Sponge-Derived Sesquiterpene Quinone, Potentiates the Antifungal Drug Caspofungin by Disrupting Hsp90 Activity and the Cell Wall Integrity Pathway. mSphere.

[140] Mahmoudi S., Rezaie S., Daie Ghazvini R., Hashemi S.J., Badali H., Foroumadi A., Diba K., Chowdhary A., Meis J.F., Khodavaisy S. (2019). In Vitro Interaction of Geldanamycin with Triazoles and Echinocandins Against Common and Emerging Candida Species. Mycopathologia.

[141] Kitson R.R.A., Kitsonova D., Siegel D., Ross D., Moody C.J. (2024). Geldanamycin, a Naturally Occurring Inhibitor of Hsp90 and a Lead Compound for Medicinal Chemistry. J. Med. Chem..

[142] Chanklan R., Aihara E., Koga S., Takahashi H., Mizunuma M., Miyakawa T. (2008). Inhibition of Ca2+-Signal-Dependent Growth Regulation by Radicicol in Budding Yeast. Biosci. Biotechnol. Biochem..

[143] Kiraz N., Şen Kaya S., Öz Y., Dağ İ. (2023). Evaluation of the Efficacy of Heat Shock Protein Inhibitors and Antifungal Drug Combinations against *Candida* spp.. Rend. Lincei.

[144] Xu L., Liu Q., Zeng Q., Wu P., Yu Q., Gu K., Xue J., Wei X. (2021). Radicicol, a Novel Lead Compound against the Migratory-Stage Schistosomula of Schistosoma Japonicum. Antimicrob. Agents Chemother..

[145] Andersen O.A., Nathubhai A., Dixon M.J., Eggleston I.M., van Aalten D.M.F. (2008). Structure-Based Dissection of the Natural Product Cyclopentapeptide Chitinase Inhibitor Argifin. Chem. Biol..

[146] Neelabh, Singh K. (2018). In-Silico Studies of Some Natural, Synthetic and Semi-Synthetic Antifungal Drugs for Their Multi-Targeting Nature. J. Microbiol. Biotechnol. Food Sci..

[147] Zhao Z., Xu Q., Chen W., Wang S., Yang Q., Dong Y., Zhang J. (2022). Rational Design, Synthesis, and Biological Investigations of N-Methylcarbamoylguanidinyl Azamacrolides as a Novel Chitinase Inhibitor. J. Agric. Food Chem..

[148] Rai M., Mares D. (2003). Plant-Derived Antimycotics: Current Trends and Future Prospects.

[149] Butassi E., Blanc A.R., Svetaz L.A. (2024). Phytolacca Tetramera Berries Extracts and Its Main Constituents as Potentiators of Antifungal Drugs against *Candida* spp.. Phytomedicine.

[150] Li C., Tu J., Han G., Liu N., Sheng C. (2022). Heat Shock Protein 90 (Hsp90)/Histone Deacetylase (HDAC) Dual Inhibitors for the Treatment of Azoles-Resistant Candida Albicans. Eur. J. Med. Chem..

[151] Li Z., Huang Y., Tu J., Yang W., Liu N., Wang W., Sheng C. (2023). Discovery of BRD4-HDAC Dual Inhibitors with Improved Fungal Selectivity and Potent Synergistic Antifungal Activity against Fluconazole-Resistant Candida Albicans. J. Med. Chem..

[152] Shahi G., Kumar M., Skwarecki A.S., Edmondson M., Banerjee A., Usher J., Gow N.A.R., Milewski S., Prasad R. (2022). Fluconazole Resistant Candida Auris Clinical Isolates Have Increased Levels of Cell Wall Chitin and Increased Susceptibility to a Glucosamine-6-Phosphate Synthase Inhibitor. Cell Surf..

[153] Wakieć R., Gabriel I., Prasad R., Becker J.M., Payne J.W., Milewski S. (2008). Enhanced Susceptibility to Antifungal Oligopeptides in Yeast Strains Overexpressing ABC Multidrug Efflux Pumps. Antimicrob. Agents Chemother..

[154] Milewski S., Mignini F., Prasad R., Borowski E. (2001). Unusual Susceptibility of a Multidrug-Resistant Yeast Strain to Peptidic Antifungals. Antimicrob. Agents Chemother..

[155] Jaromin A., Zarnowski R., Markowski A., Zagórska A., Johnson C.J., Etezadi H., Kihara S., Mota-Santiago P., Nett J.E., Boyd B.J. (2024). Liposomal Formulation of a New Antifungal Hybrid Compound Provides Protection against Candida Auris in the Ex Vivo Skin Colonization Model. Antimicrob. Agents Chemother..

[156] Espinel-Ingroff A., Wiederhold N.P. (2024). A Mini-Review of In Vitro Data for Candida Species, Including C. Auris, Isolated during Clinical Trials of Three New Antifungals: Fosmanogepix, Ibrexafungerp, and Rezafungin. J. Fungi.

[157] Ghannoum M., Arendrup M.C., Chaturvedi V.P., Lockhart S.R., McCormick T.S., Chaturvedi S., Berkow E.L., Juneja D., Tarai B., Azie N. (2020). Ibrexafungerp: A Novel Oral Triterpenoid Antifungal in Development for the Treatment of Candida Auris Infections. Antibiotics.

[158] Nunnally N.S., Etienne K.A., Angulo D., Lockhart S.R., Berkow E.L. (2019). In Vitro Activity of Ibrexafungerp, a Novel Glucan Synthase Inhibitor against *Candida glabrata* Isolates with FKS Mutations. Antimicrob. Agents Chemother..

[159] Wiederhold N.P., Najvar L.K., Olivo M., Morris K.N., Patterson H.P., Catano G., Patterson T.F. (2021). Ibrexafungerp Demonstrates in Vitro Activity against Fluconazole-Resistant Candida Auris and in Vivo Efficacy with Delayed Initiation of Therapy in an Experimental Model of Invasive Candidiasis. Antimicrob. Agents Chemother..

[160] Phillips N.A., Rocktashel M., Merjanian L. (2023). Ibrexafungerp for the Treatment of Vulvovaginal Candidiasis: Design, Development and Place in Therapy. Drug Des. Devel. Ther..

[161] Thompson G.R., Soriano A., Cornely O.A., Kullberg B.J., Kollef M., Vazquez J., Honore P.M., Bassetti M., Pullman J., Chayakulkeeree M. (2023). Rezafungin versus Caspofungin for Treatment of Candidaemia and Invasive Candidiasis (ReSTORE): A Multicentre, Double-Blind, Double-Dummy, Randomised Phase 3 Trial. Lancet.

[162] Pappas P.G., Vazquez J.A., Oren I., Rahav G., Aoun M., Bulpa P., Ben-Ami R., Ferrer R., Mccarty T., Thompson G.R. (2023). Clinical Safety and Efficacy of Novel Antifungal, Fosmanogepix, for the Treatment of Candidaemia: Results from a Phase 2 Trial. J. Antimicrob. Chemother..

[163] Fujita K.I., Irie M., Ping X., Taniguchi M. (1999). Antifungal Activity of Radicicol against Mucor Flavus IFO 9560. J. Biosci. Bioeng..

[164] Escalante A., Gattuso M., Pérez P., Zacchino S. (2008). Evidence for the Mechanism of Action of the Antifungal Phytolaccoside B Isolated from Phytolacca Tetramera Hauman. J. Nat. Prod..

